# The Mineral Waters of the United States and Their Therapeutic Uses

**Published:** 1899-10

**Authors:** 


THE MINERAL WATERS OF THE UNITED STATES AND THEIR THERAPEUTIC USES. With an account of the various mineral spring localities, their advantages as health resorts. By JAMES K. CROOK, A. M., M. D., New York. New York and Philadelphia: Lea Brothers & Co. 1899.

The efficacy of water in the treatment of disease was known and practised even in the days when Rome was in her glory and down the ages without interruption have come the blessings and advantages of the most important of nature's elements. European peoples have not failed to appreciate the immense advantages of their natural resources of this character and the most successful physicians are those who send the majority of their patients to some fashionable watering place for rest, recuperation, and the internal and external use of water. Perhaps the change of scenery, of surroundings, and of habits have much to do with the results obtained, but certain it is that patients at these spas will use water plentifully—whereas at home they hardly know the color, taste or feeling of water. Americans have lately been in the habit of going to Carlsbad, Ems, Baden Baden and other European springs, when in fact within their own borders are found the close counterparts of the best foreign springs. The absence of knowledge regarding our own springs may have been responsible for this to a certain degree and the work in question will undoubtedly do much to satisfy this want. Though several excellent volumes have been written they have become antiquated through natural causes, such as the abandonment of certain once popular spring resorts and the development of a large number of new and important spas. In the present volume more than 200 mineral spring localities are for the first time described in a book of this kind.

The author divides his work into two parts, viz.: mineral waters and their therapeutic uses and the mineral springs and wells of the United States, with the topographical and climatic features of each state and territory. In part first the author considers the question of hydriatrics in general, the origin of springs and their sources of mineralisation; the classification of mineral springs; the solid and gaseous components of mineral waters; baths and douches and their medicinal use, and the therapeutics of mineral waters.

In Part II. the author gives the analyses of the different mineral springs from Maine to California and from Alaska to Florida, with their medicinal qualities. He has made use of the elaborate and well-nigh exhaustive tabulation of the American mineral springs, made for the United States geological survey, by Dr. Albert C. Peale, besides carefully examining all available literature on the subject. Moreover, letters of inquiry have been addressed to every spring resort and commercial spring and much valuable information has been gleaned by personal visits from time to time to many important localities in the different states. It is thus very evident that the author has made a most careful examination and enumeration of these waters, and to the physician in search of certain waters this volume will be of the greatest aid. The book is well printed and nicely bound.

